# Meta-analysis of predictive symptoms for Ebola virus disease

**DOI:** 10.1371/journal.pntd.0008799

**Published:** 2020-10-23

**Authors:** Vageesh Jain, Andre Charlett, Colin S. Brown

**Affiliations:** 1 North East and North Central London Health Protection Team, Public Health England, London, United Kingdom; 2 Institute for Global Health, University College London (UCL), London, United Kingdom; 3 National Infection Service, Public Health England, London, United Kingdom; 4 King’s Sierra Leone Partnership, King’s Centre for Global Health, King’s Health Partners and King’s College London, London, United Kingdom; 5 Department of Infection, Royal Free London NHS Foundation Trust, London, United Kingdom; Faculty of Science, Ain Shams University (ASU), EGYPT

## Abstract

**Introduction:**

One of the leading challenges in the 2013–2016 West African Ebola virus disease (EVD) outbreak was how best to quickly identify patients with EVD, separating them from those without the disease, in order to maximise limited isolation bed capacity and keep health systems functioning.

**Methodology:**

We performed a systematic literature review to identify all published data on EVD clinical symptoms in adult patients. Data was dual extracted, and random effects meta-analysis performed for each symptom to identify symptoms with the greatest risk for EVD infection.

**Results:**

Symptoms usually presenting late in illness that were more than twice as likely to predict a diagnosis of Ebola, were confusion (pOR 3.04, 95% CI 2.18–4.23), conjunctivitis (2.90, 1.92–4.38), dysphagia (1.95, 1.13–3.35) and jaundice (1.86, 1.20–2.88). Early non-specific symptoms of diarrhoea (2.99, 2.00–4.48), fatigue (2.77, 1.59–4.81), vomiting (2.69, 1.76–4.10), fever (1.97, 1.10–4.52), muscle pain (1.65, 1.04–2.61), and cough (1.63, 1.24–2.14), were also strongly associated with EVD diagnosis.

**Conclusions:**

The existing literature fails to provide a unified position on the symptoms most predictive of EVD, but highlights some early and late stage symptoms that in combination will be useful for future risk stratification. Confirmation of these findings across datasets (or ideally an aggregation of all individual patient data) will aid effective future clinical assessment, risk stratification tools and emergency epidemic response planning.

## Author summary

Ebola is a rare but deadly virus, found mainly in sub-Saharan Africa. People can get it through direct contact with an infected animal (bat or nonhuman primate) or a sick or dead person infected with Ebola virus. The virus can cause a host of different symptoms that overlap with many other diseases, making diagnosis a challenge for doctors, particularly with a lack of available rapid diagnostic tests or laboratory facilities.

This meta-analysis of predictive symptoms for Ebola virus shows that patients initially presenting with symptoms usually found late in illness, including confusion, conjunctivitis, dysphagia and jaundice were significantly more likely to end up with a diagnosis of Ebola. Early symptoms (typically not thought to be specific to Ebola), including diarrhoea, fatigue, vomiting, fever, muscle pain and cough were also found to be more predictive of a diagnosis of Ebola virus.

The existing literature fails to provide a unified position on the symptoms most predictive of Ebola. Confirmation of our findings will aid effective future clinical assessment, risk stratification tools and emergency epidemic response planning.

## Introduction

One of the leading challenges in the 2013–2016 West African Ebola virus disease (EVD) outbreak was how best to quickly identify patients with EVD, separating them from those without the disease. The early symptoms of EVD are similar to other diseases endemic in African regions, including malaria and gastroenteritis. Patients presenting with various non-specific symptoms made it difficult to contain the outbreak, and keep health facilities open and functioning, due to limited levels of infection prevention and control (IPC), diagnostic infrastructure, and dedicated staff needed to screen, isolate, and test all patients with a compatible syndrome [1]. Current gold standard diagnosis of EVD requires reverse transcription polymerase chain reaction (RT-PCR) testing of venous blood samples in a laboratory setting [2]. This diagnostic process is lengthy, particularly in countries with weak healthcare and laboratory infrastructure. In the initial phase of the West African outbreak there was limited capacity to test for EVD and blood samples needed transport infrastructure to newly created laboratories for RT-PCR testing [3]. Sociocultural barriers to healthcare further compounded the difficult situation resulting in late presentations, highlighting the need for optimal clinical risk prediction and diagnostic tools [4]. The capacity to safely isolate all suspect patients while awaiting RT-PCR results, and therefore protect local healthcare staff and keep health facilities operational, was very limited in the early stages of outbreak response [5].

The current Centers for Disease Control and Prevention (CDC) clinical case definition for EVD includes someone who has a fever or other symptoms (severe headache, fatigue, muscle pain, vomiting, diarrhoea, abdominal pain or unexplained haemorrhage) and an epidemiological risk factor in the preceding 21 days [6]. Aside from contact with probable cases, the World Health Organization (WHO) suspect case definition [7] includes sudden onset fever plus at least three of headaches, anorexia/loss of appetite, stomach pain, vomiting, diarrhoea, lethargy, muscle aches, joint pain, dysphagia, difficulty breathing and hiccups. Given the increase in clinical experience resulting from the 2013–16 EVD outbreak, constructing a more specific constellation of symptoms diagnostic of EVD may be possible.

The existing literature explores the predictive value of individual and groups of symptoms for EVD, but often fails to reach consistent conclusions or does not compare symptoms across those with and without the disease [8–11]. We therefore aimed to conduct a systematic review and meta-analysis to aggregate available data on symptom predictors for EVD, to see if we could provide clinicians with a more robust guide to the most specific clinical features of EVD across several countries and settings. This would enable quicker diagnosis of probable cases in resource-limited settings, and earlier stratification of patients as high-risk, permitting appropriate clinical and public health precautions to be taken to counteract the spread of disease.

## Methods

### Retrieval of studies

Identification of relevant existing literature was performed by an online search in MEDLINE and EMBASE for studies published from 1^st^ January 1946 to 3^rd^ July 2020. The MESH headings (keywords) searched were “ebola” and “symptom*” or “clinical” or “predict*” or “suspect*”. Two independent reviewers (VG, CB) screened the list of title and abstracts, and the full text of chosen manuscripts was analysed. Disagreements were discussed until consensus was achieved. Data on symptom presentation was extracted and entered into an Excel spreadsheet. In addition to the MEDLINE/EMBASE search, citation tracking of articles was used to identify any remaining studies. Reference lists of review articles were also examined. Unpublished data was not reviewed due to uncertain data quality.

### Inclusion and exclusion criteria

All studies evaluating individual symptoms in predicting EVD diagnosis were included. All study designs from any year in an adult population (including the elderly) were eligible, except case reports/case series, which did not fulfill the inclusion criteria because of an inability to compare the symptoms in both EVD and non-EVD patients. To avoid selection bias, no subjective quality criteria were applied to the studies for inclusion. Exclusion criteria included: (1) studies of exclusively pediatric or pregnant patients, due to the varying presentation of EVD in these groups, (2) insufficient data on symptoms before diagnosis in either EVD or non-EVD groups, (3) EVD strains other than Zaire virus (EBOV) (4) no original data on symptoms in an EVD cohort e.g. literature reviews, and (5) studies that were not written in English or French (although no French studies were found), due to practical limitations with translation.

### Data extraction

From each study the author details, country of population studied, and year of publication were recorded. Study design, sample size, gender and age demographics were ascertained. The numbers of cases with and without each symptom of EVD after definitive diagnostic testing were extracted for each study. All patients recorded as testing negative for EVD were screened in the same facilities as those recorded as positive. Pooled odds ratios (OR) and 95% confidence intervals were calculated from this information. In one study [9], proportions of cases and non-cases with symptoms were not provided, but were back calculated from the reported sensitivity and specificity. Four studies also reported p-values for the predictive value of individual symptoms [8, 10, 12, 13]. In all cases, if multivariable analysis was undertaken, the variables controlled for were extracted from the text. Five studies [9, 12–15] also investigated the area under the Receiver Operating Characteristic (ROC) curve to understand the usefulness of their clinical predictive models, and these data were recorded where available.

### Statistical analysis

For each symptom investigated, numbers of EVD and non-EVD patients were aggregated across included studies. We employed a random effects meta-analysis in STATA [16] using the “metan” command for each specific symptom. Random effects were used to account for between study heterogeneity [17]. This provided a pooled odds ratio (pOR), 95% confidence intervals and a p-value, for each symptom (Table 3); with a summary plot for all symptoms presented in the supplementary data (S1 Fig). Symptoms presented in this analysis were investigated in at least 5 out of the 8 studies and heterogeneity between studies was estimated with Tau-squared. Very similar symptoms (such as painful swallowing and difficulty swallowing) were combined for the purposes of meta-analysis.

## Results

**Fig 1:**
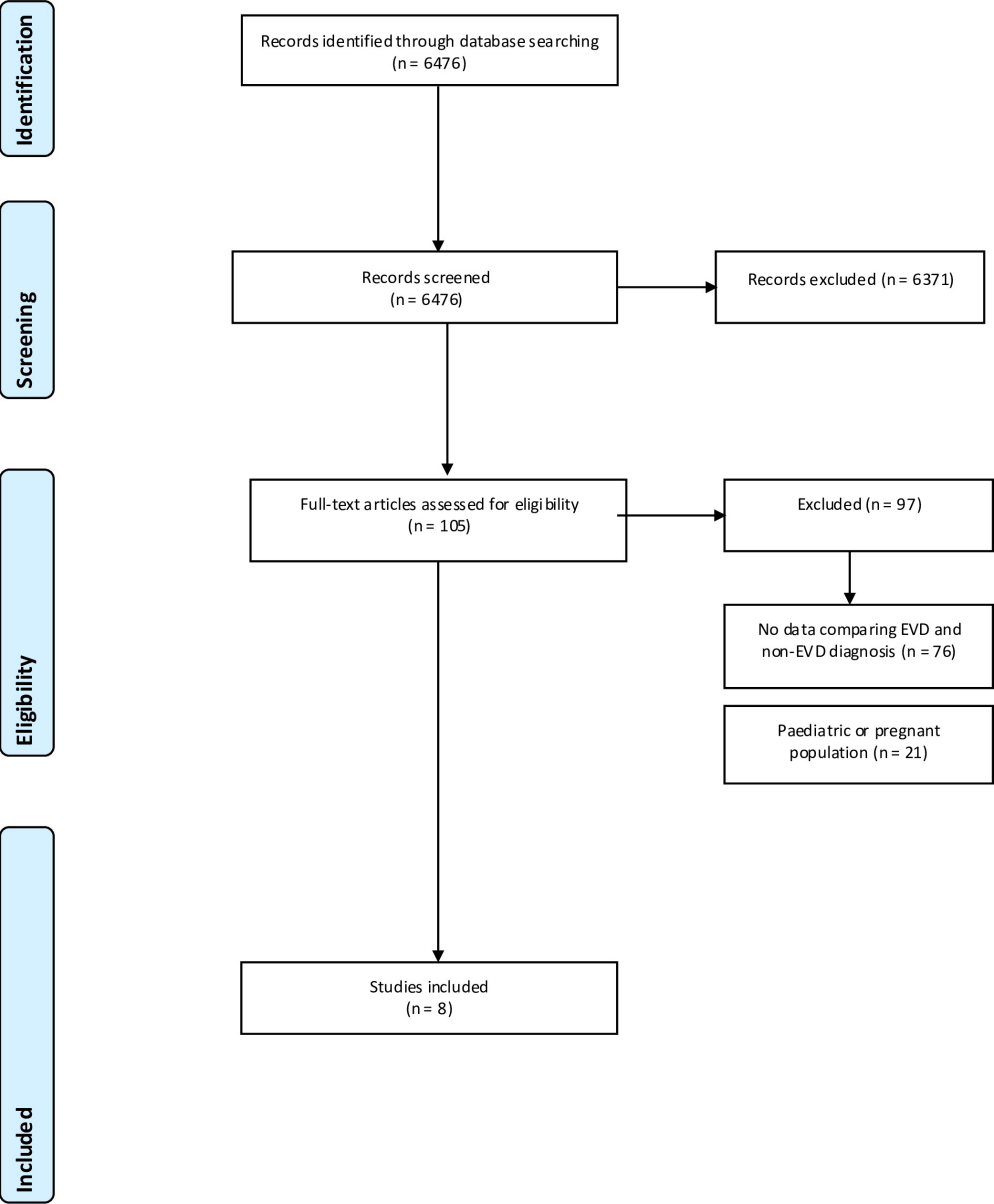
PRISMA flow diagram.

The PRISMA flow diagram (Fig 1) illustrates the process for selection of papers in this study.

### Literature search

The initial search on MEDLINE and EMBASE produced 6476 results, as shown in Fig 1. After applying exclusion criteria, there were 105 papers met our criteria from title and abstract analysis. On further review, 76 of these studies did not compare proportions of patients with symptoms in laboratory confirmed EVD cases with non-EVD, primarily investigating predictors for mortality among EVD survivors and those who died [18–22]. A further 21 papers were excluded as they exclusively investigated either a paediatric or pregnant population. This left a total of eight studies [8–10, 12–15, 23] for meta-analysis.

### Description of included studies

Table 1 shows all included studies, details on study populations, and predictive symptoms for EVD. All are retrospective cohort studies in design, conducted between 2014 and 2015 during the West African 2013–2016 EVD outbreak. Lu et al conducted the largest study with 1014 participants (excluding those with no known PCR result), whilst Arranz et al included just 75 participants in their study. The number of symptoms investigated varies from 13 in one study [9] to 23 in others [8, 10].

**Table 1:** Studies investigating the predictive value of symptoms for EVD.

Study	Year and location	Design	Population (n)	EVD cases (N)	Predictive Symptoms for EVD (Crude OR, 95% CI)	Number of Symptoms Investigated	Median Duration of Symptoms at Presentation in days (IQR)	Other Variables Adjusted For	Predictive Model Area under ROC curve^a^ (95% CI)	Risk of Bias^b^
Arranz et al [10]	December 2014-March 2015, Sierra Leone	Retrospective cohort	n = 75, 67% male 50.3% median age = 34	31	Fatigue (22.8, 2.8–182.4 p<0.001), diarrhoea (5.0, 1.8–13.5 p = 0.002), muscle pain (3.8, 1.4–9.9 p = 0.009), vomiting (3.3, 1.3–8.7 p = 0.018) (dysphagia, bleeding p <0.001 –no OR)	23	Non-EVD = 2 (1–6) EVD = 3 (1–5)	Univariable analysis	-	A, B, D, E
Lu et al [23]	September-November 2014, Sierra Leone	Retrospective cohort	n = 1014, 51% male, median age = 26	563	All except haemorrhage more frequent in EVD compared to non-EVD (p<0.05)—ORs not reported	22	EVD = 5 (3–7)	-	-	A, B, D, E
Gao et al [15]	October 2014 –March 2015, Sierra Leone	Retrospective cohort	n = 773 [Population breakdown unpublished]	285	Vomiting (2, 1.5–2.7), diarrhoea (1.94, 1.44–2.61), weakness (1.83, 1.27–2.63), confusion (3.03, 1.62–5.64), loss of appetite (2.98, 2.12–4.20), conjunctivitis (1.89, 1.34–2.66), hiccups (2.77, 1.82–4.23)	18	-	Univariable analysis (then multivariable for significant symptoms)	Seven symptom model (co-prescence of 3 or more of: vomiting, diarrhoea, weakness, loss of appetite, conjunctivitis, hiccups and confusion) 0.728 (0.691–0.765)	A, B,D,E
Lado et al [8]	May 2014- December 2014, Sierra Leone	Retrospective cohort	n = 724, 56% male, median age = 31	464	Vomiting (1.6, 1.1–2.1 p = 0.006), diarrhoea (1.5, 1.1–2 p = 0.022), fatigue (1.7, 1.2–2.4 p = 0.001), conjunctivitis (2.6, 1.7–3.9 p<0.0005), hiccups (1.9, 1.1–3.5 p-0.026), confusion (2.8, 1.7–4.7 p<0.0005), no joint pain (0.6, 0.4–0.9 p = 0.017), no headache (0.7, 0.5–1.0 p = 0.045)	23	Non-EVD = 4 (2–7) EVD = 4 (3–6)	Univariable analysis	-	A, B, D, E
Levine et al [9]	September 2014- January 2015, Liberia	Retrospective Cohort	n = 382, 52% male, median age = 31.5	160	Diarrhoea (2.62, 1.6–4.3), anorexia (2.12, 1.25–3.6), muscle pain (1.87, 1.16–3), difficulty swallowing (1.86, 1.09–3.18), no abdominal pain (0.52, 0.31–0.87)	13	Non-EVD = 3 (0–18) EVD = 3 (1–11)– 95% CI	Univariable analysis (then multivariable for significant symptoms and epidemiological risk factor)	Six variable model (sick contact, diarrhoea, loss of appetite, muscle pain, dysphagia, absence of abdominal pain) 0.75 (0.7–0.8)	A, B
Loubet et al [14]	December 2014-Feb 2015, Guinea	Retrospective Cohort	n = 145, 48% male, median age = 29	76	Fever (14.6, 5.3–40.6 p<0.001), anorexia (3.0, 1.5–6.1 p = 0.002), diarrhoea (2.9, 1.4–6.0 p = 0.003), intense fatigue (2.3, 1.2–4.5 p = 0.02), vomiting (2.1, 1.1–4.2 p = 0.04)	14	Non-EVD = 2 (1–5) EVD = 3 (1–5)	Univariable analysis (then multivariable for significant symptoms and epidemiological risk factor)	Three variable model (fever>38.5°C, anorexia and presence of risk factor) 0.85 (0.78–0.91)	A, B, E
Oza et al[13]	November 2014 –March 2015, Sierra Leone	Retrospective cohort	n = 424, 50% male, median age = 26	252	Univariable: Diarrhoea (1.06, 0.66–1.47 p<0.001) Multivariable: Conjunctivitis (1.44, 0.93–1.95 p<0.001), Diarrhoea (1.11, 0.6–1.61 p<0.001)	14	Non-EVD = 3 (2–6) EVD = 3 (2–5)	Univariable analysis (then multivariable for significant symptoms and lab blood tests)	Six symptom model (headache, diarrhoea, SOB^c^, nausea/vomiting, loss of appetite and conjunctivitis) 0.84 (0.79–0.86)	A, B
Yan et al [12]	October-November 2014, Sierra Leone	Retrospective Cohort	n = 154, 57% male, mean age = 28	108	All symptoms (p<0.001)–no ORs reported	21	EVD = 5.8+/-3.3	-	Number of symptoms at admission (cut-off of six symptoms) 0.789 (0.715–0.862)	A, B, D, E

a ROC = Receiver Operating Characteristic Curve

^b^ A: selection, B: measurement, C: detection, D: reporting, E: other

^C^ Shortness of breath

Although two studies did not provide a gender or age breakdown of included participants [8, 15], Table 2 displays the age and gender of both EVD and non-EVD patients, after aggregating all remaining studies. The median age was 28.6 years for EVD patients and 31.4 years for non-EVD patients.

**Table 2:** All Studies EVD vs. non-EVD.

	EVD, N = 1939 (%)	Non-EVD, N = 1752 (%)
Median age (years)	28.6	31.4
Male	582 (30.0)	567 (32.4)
Female	608 (31.4)	437 (24.9)

* Excluded: age unavailable for Gao et al [15], Lado et al [8], Lu et al[23], gender unavailable for Gao et al and Lado et al

### Risk of bias

To determine risk of bias for individual studies included in this analysis, an adaptation of the Scottish Intercollegiate Guidelines Network (SIGN) critical appraisal checklist for cohort studies [24] was used alongside relevant components of the Cochrane Collaboration’s Risk of Bias tool [25]. Table 1 shows which studies contained methodological flaws reflecting the risk of bias (selection, measurement, detection, reporting, and other sources of bias). The criteria used in our assessment are further described in the supplementary data (S1 Table).

In all studies [8–10, 12–15, 26] some or all patients were referred to hospitals/EVD treatment units from community outreach teams, who performed an initial screening for suspect EVD cases based on the WHO case definition. In one study a similar screening tool was developed and used for assessment on arrival at hospital [8]. Although the initial screening of patients outside of the clinical environment could have introduced selection bias, the use of standardized case definitions minimised this. There was significant potential for measurement bias in all studies, since factors that could have affected symptom reporting such as concurrent medication use, were not routinely and consistently collected. One study [12] used symptoms that were recorded by patients (yes, no, unknown) in a self-reported questionnaire, for subsequent analysis. In all remaining studies, clinical professionals recorded symptoms on admission, to minimise measurement bias. Detection bias was minimised in all studies by carrying out EBOV RT-PCR tests in included study participants, to stratify into EVD and non-EVD groups, although it was not possible to mask the final diagnosis to researchers analysing the data retrospectively. Reporting bias was widely prevalent, specifically due to a lack of adequately identifying and adjusting for confounding factors in the symptom predictor analysis. Four studies [9, 13–15] conducted a multivariable analysis, adjusting for the presence of EVD contact in the previous 21 days [9], the presence of any EVD epidemiological risk factor [14], the presence of multiple symptoms together [15] and multiple symptoms with biochemical lab test results [13]. Three of the remaining studies [8, 10, 26] provided odds ratios with 95% confidence intervals, but two failed to do this [12, 23], reporting proportions of patients with symptoms and p-values.

### Symptom analysis

Table 3 shows the odds ratios and 95% confidence intervals for the individual symptoms that were investigated in at least five of the included studies. The number of studies in which each symptom was investigated, the prevalence of each symptom in EVD and non-EVD groups, as well the odds ratio, 95% confidence interval and p-value for each symptom in EVD compared with non-EVD are also displayed. A p-value of ≤0.05 was used as a marker for evidence of association.

**Table 3:** Estimated pOR from random-effects meta-analysis for each symptom.

Symptom	Pooled Odds Ratio (95% CI)	P-Value	Prevalence in EVD patients (%)	Prevalence in non-EVD patients (%)	Number of studies (n = 8)	Tau-squared
Confusion	3.04 (2.18–4.23)	<0.001	22.6	7.7	5	0.04
Diarrhoea	2.99 (2.00–4.48)	<0.001	53.6	30.0	8	0.27
Conjunctivitis	2.90 (1.92–4.38)	<0.001	25.3	9.3	7	0.19
Fatigue	2.77 (1.59–4.81)	<0.001	59.6	36.9	8	0.51
Vomiting	2.69 (1.76–4.10)	<0.001	31.0	15.4	8	0.30
Anorexia	2.02 (0.78–5.28)	0.15	73.2	57.8	6	1.35
Fever	1.97 (1.10–3.52)	0.02	82.0	69.8	8	0.58
Dysphagia	1.95 (1.13–3.35)	0.02	20.8	12.8	7	0.37
Sore throat	1.88 (0.87–4.07)	0.11	22.1	10.8	5	0.53
Jaundice	1.86 (1.20–2.88)	0.005	51.8	39.8	5	0.09
Hiccups	1.82 (0.93–3.56)	0.08	17.9	16.3	7	0.61
Muscle Pain	1.65 (1.04–2.61)	0.03	21.4	18.1	8	0.36
Cough	1.63 (1.24–2.14)	<0.001	4.5	2.4	6	0.04
Bleeding	1.51 (0.86–2.67)	0.16	31.9	34.3	7	0.25
Chest pain	1.50 (0.88–2.54)	0.14	56.0	45.8	5	0.28
Abdominal Pain	1.47 (0.97–2.21)	0.07	51.1	43.3	8	0.28
Headache	1.34 (0.85–2.11)	0.21	24.7	17.2	8	0.36
Joint Pain	1.29 (0.85–1.97)	0.23	32.3	27.2	8	0.30
Shortness of Breath	1.02 (0.41–2.54)	0.97	49.5	55.7	7	1.18

**Fig 2:**
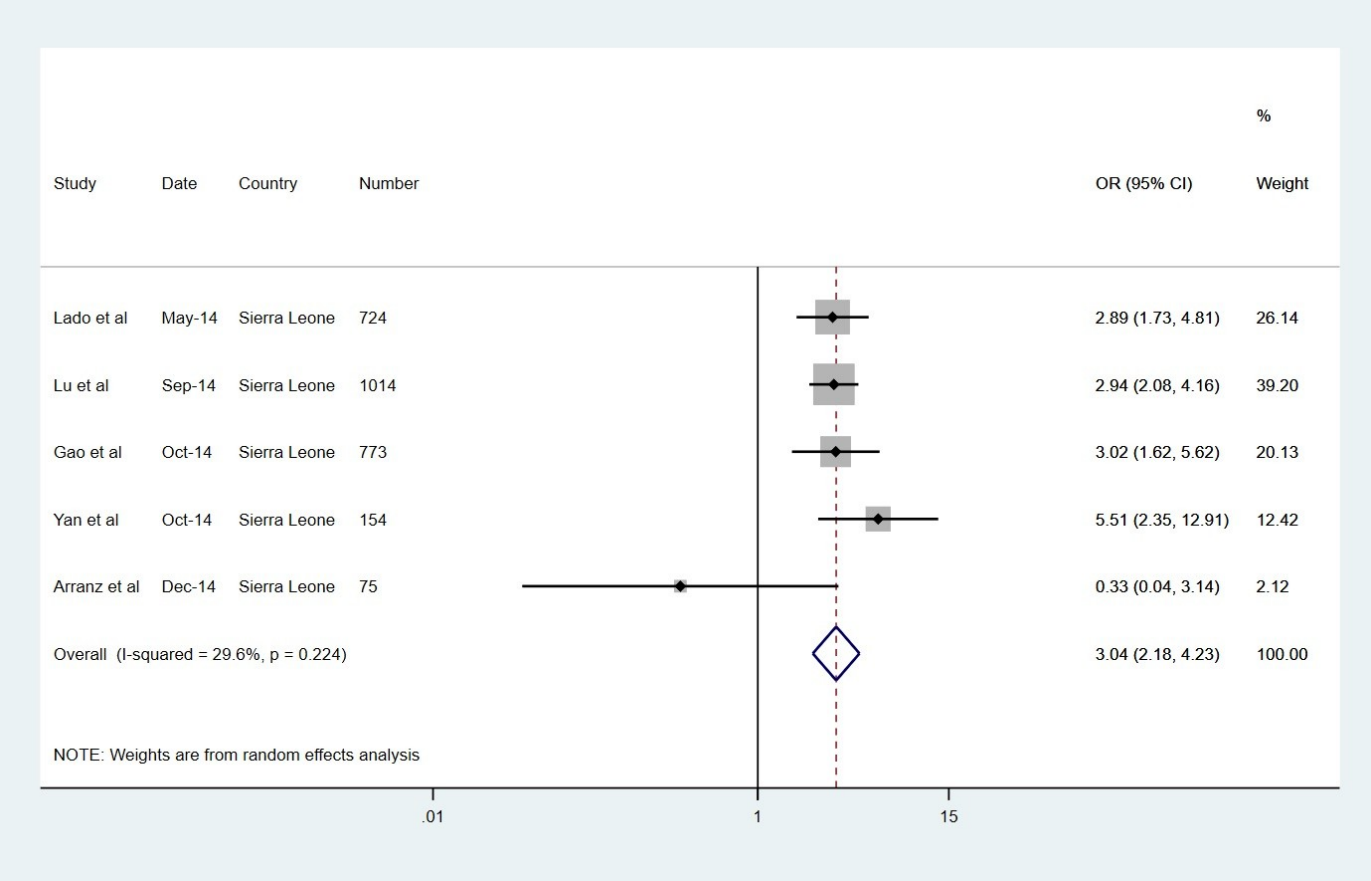
Symptoms with the largest pooled odds ratio estimates for of EVD, CONFUSION.

**Fig 3:**
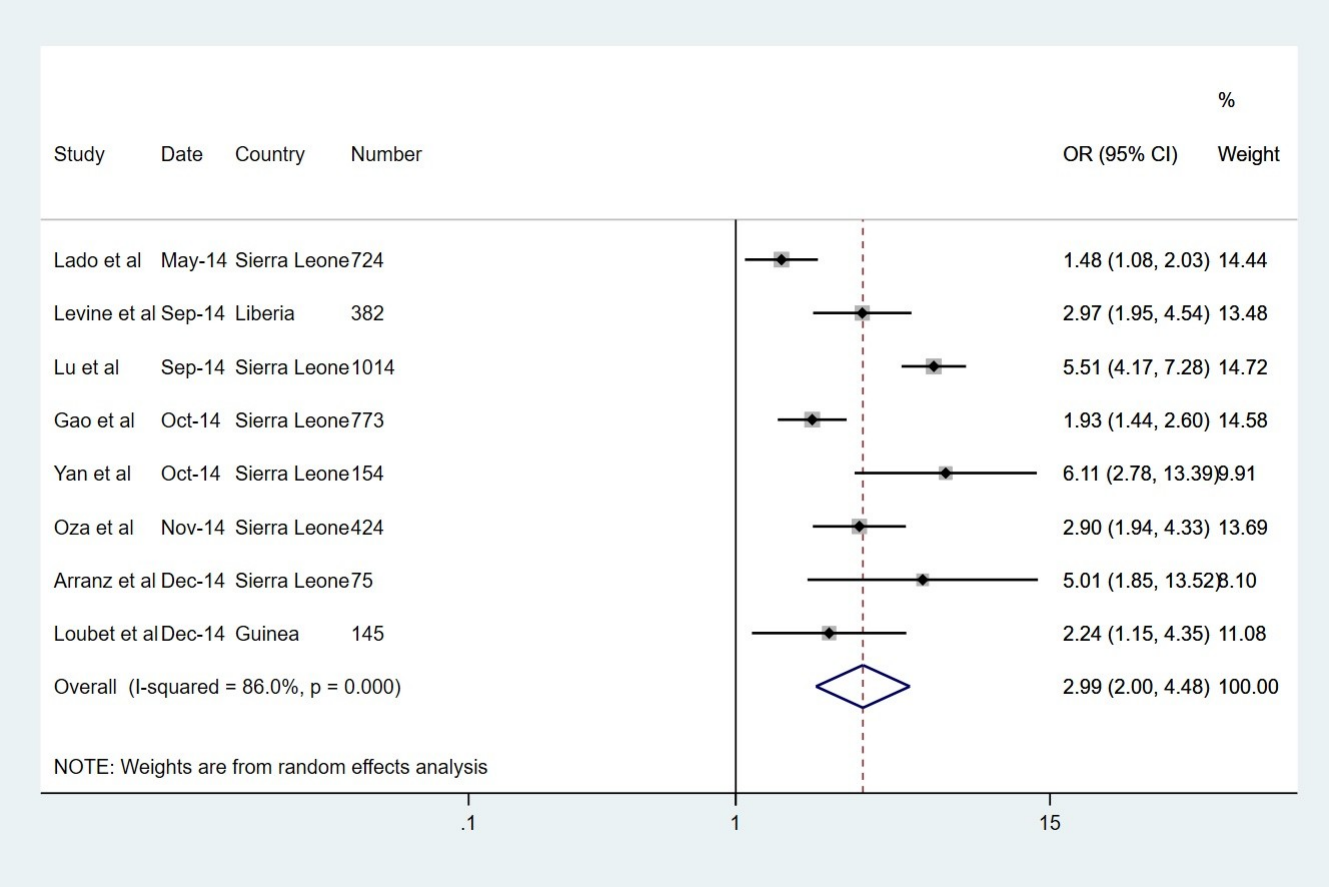
Symptoms with the largest pooled odds ratio estimates for of EVD, DIARRHOEA.

**Fig 4:**
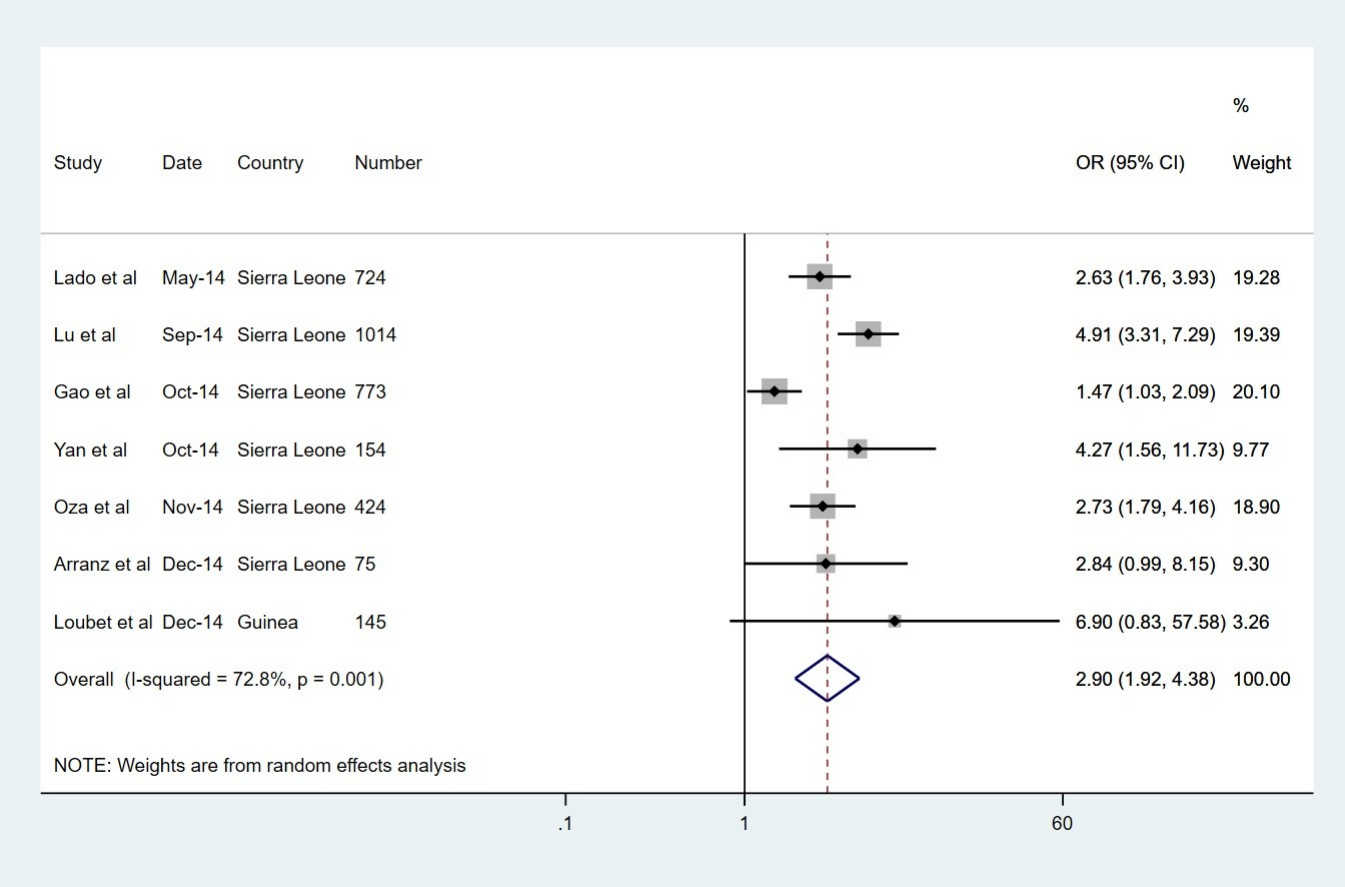
Symptoms with the largest pooled odds ratio estimates for of EVD, CONJUNCTIVITIS.

All symptoms except bleeding and shortness of breath were more prevalent in the EVD group. The most common symptoms in the EVD group were fever (82%) and anorexia (73%), but these were also the most common symptoms in the non-EVD group. Figs 2–4 display forest plots for the three most predictive symptoms: confusion (pOR 3.04, 95% CI 2.18–4.23), diarrhoea (2.99, 2.00–4.48) and conjunctivitis (2.90, 1.92–4.38). The weighting given to each study (based on sample size) is also presented alongside. Other strongly predictive symptoms included fatigue (2.77, 1.59–4.81), vomiting (2.69, 1.76–4.10), fever (1.97, 1.10–3.52), dysphagia (1.95, 1.13–3.35), jaundice (1.86, 1.20–2.88), muscle pain (1.65, 1.04–2.61), and cough (1.63, 1.24–2.14), also displaying evidence of association. There was some weaker evidence of association for hiccups (OR 1.82, 0.93–3.56) and abdominal pain (1.47, 0.97–2.21). The remaining symptoms of anorexia, bleeding, headache, shortness of breath, chest pain, joint pain and sore throat, were not significantly associated with EVD. Notably there was a very high level of statistical heterogeneity for both anorexia and shortness of breath, with high tau-squared values.

## Discussion

### Key findings

Confusion was the symptom most predictive for EVD (pOR 3.04, 95% CI 2.18–4.23), with patients presenting with confusion being three times more likely to have a diagnosis of EVD than not. Other specific symptoms thought to present late in illness [27] were also strongly associated with EVD, including conjunctivitis, dysphagia and jaundice. Early non-specific symptoms of diarrhoea, vomiting, fatigue, fever, muscle pain and cough were also strongly associated with EVD.

### Building on individual studies

Our analysis echoes previous findings [8, 15] to an extent. But contrary to these studies, we found fever, cough and muscle pain were also predictive for EVD. Gao et al [15] found cough and muscle pain were significant on univariable analysis, but not after adjusting for multiple symptoms. Our result for fever was likely positively skewed due to one study [9] that reported a very high odds ratio (OR 14.6, 5.26–40.6), unlike other studies. Fever is a contested symptom in the literature, with two studies [12, 14] reporting it as a strongly predictive symptom, but other larger studies not finding this to be the case[8, 9, 13, 15]. This specific heterogeneity may be in part due to the fact that fever occurrence can vary over the course of a day meaning that rates of reported fever may have varied due to time of measurement, and history of fever is a subjective symptom prone to misreport.

Negative predictors are also important considerations in the context of diagnosis. Lado et al [8] found that no joint pain (OR 0.6, 0.4–0.9), and no headache (OR 0.7, 95% CI 0.5–1.0) were predictive for EVD. Levine et al [9] found the same for lack of abdominal pain (OR 0.52, 0.31–0.87). Our analysis shows these findings are not consistent among studies, with none of these when pooled were found to be negatively predictive for EVD.

Many reasons may exist for the variation between the findings of different studies. Firstly, varying definitions for symptoms, or differing measurement practices, for instance how to measure confusion or when exactly temperature was recorded, may have played a role. Regardless of study protocol, some symptoms cannot be objectively recorded and rely on subjective measures, introducing an element of measurement error and bias in all studies like this. Secondly, the time between onset of symptoms and presentation to health services will have affected where patients were in their EVD presentation, leading to different conclusions on predictive symptoms. Despite this, four of the included papers showed that the duration of symptoms for both EVD and non-EVD groups were similar [8–10, 14]. Thirdly, population factors may have altered the clinical presentation of EVD from one study to another. For instance, the average age of patients, the gender mix, regional patterns in outbreaks of infectious disease other than EVD, and sociocultural differences in illness reporting could have affected the results of individual studies. Finally, due to the nature of an EVD outbreak, rigorous checks and protocols on study methodology may have been difficult to develop and adhere to, particularly toward the start of the outbreak, limiting the quality of data collection and increasing the likelihood of bias.

### Limitations

The most significant limitation of this study is the lack of multivariable analysis to account for the presence of different symptoms or multiple symptoms presenting together. Although a database that enables linkage of individual patient records has emerged recently[28], four of the studies in this review are not currently included. Our univariable analysis is valuable in evaluating the most specific individual symptoms for Ebola, and represents the next best alternative to a multivariable analysis, given the non-availability of individual level data. It is potentially possible to attempt network meta-analysis [29] restricted to the two studies that undertook multivariable analysis, although the assumption of consistency is more likely to be violated in observational studies of subjective symptoms than in clinical trials of treatments. Therefore, this has not been attempted.

Secondly, we were not able to account for the timing of presentation in the statistical analysis. If a patient presented after many days of illness, this may have led to different presenting symptoms, compared with an early admission to a treatment unit. However, Table 1 shows information from individual studies on median duration of symptoms at presentation, which appears similar between Ebola and non-Ebola cases. It is therefore unlikely that the duration of symptoms before presentation would have biased the overall results, as both groups reportedly presented at similar times after symptom onset, though this relies on the accuracy of recall.

Thirdly, no data was recorded prospectively given that time constraints and outbreak response clearly took priority. The emergency context may also have led to selection bias in that initial screening criteria determined who was tested (based on suspicious symptoms), and therefore included in studies.

Finally, age and gender were not adjusted for in the statistical analysis, meaning these factors could have accounted for disparities in symptoms between EVD and non-EVD groups. However, Table 2 shows median ages as well as the proportions of participants who were male and female, in both aggregated EVD and non-EVD groups. The proportion of males is slightly higher in the non-EVD group, but the median age is similar between both groups, and would therefore be an unlikely source of confounding.

### Implications for clinical practice

Successful management of EVD epidemics requires appropriate mechanisms through which to identify suspect cases for isolation and further testing with limited resources. This is not only a clinical imperative, to help prioritise treatment and allocation of scarce clinical resource based on need, but also from a public health perspective this can minimise the potential risk of onward transmission of disease through unnecessary admissions [30]. Though in practice this transmission has been successfully minimised [31]. Clinical risk stratification also helps to maintain adequate bed capacity and prevent systems from being overwhelmed, which is key to epidemic control, particularly in the initial phases of outbreak response[3].

Although blood tests can be performed to diagnose EVD, in resource limited settings laboratory services may not be in close proximity, and transport links may be weak. Patients with symptoms for fewer than three days will generally still have to wait for a second negative test result before discharge [32]. Thus the first few days after presentation to healthcare services are critical, and depend upon high quality clinical screening and management.

In general, symptoms we found to be most predictive for EVD at presentation confirm findings from previous studies, with a few exceptions. Our results clarify some of the disagreement found between existing studies, and provide a broader picture of EVD presentation. It is apparent that not only late-onset symptoms are strongly predictive for EVD, but also earlier-onset ones such as diarrhoea, vomiting, fatigue and fever.

### Implications for future research

Future case report forms should aim to record sources of confounding including medication taken by the patient, which may mask symptoms such as fever or pain. Studies should aim to include such factors in multivariable analysis of symptoms, to provide an understanding of the set of symptoms that are best able to diagnose PCR confirmed EVD. There is also a wide heterogeneity in the range and choice of symptoms investigated, as well as how they are measured or defined, in existing studies. For instance, Levine et al [9] did not include confusion or conjunctivitis in their investigated symptoms, whilst others did and found these symptoms to be correlated with a diagnosis of EVD [8, 15]. Oza et al grouped joint and muscle pain, and loss of appetite and anorexia, together[13], whilst remaining studies considered these symptoms separately. Where possible, a full range of agreed individual symptoms should be included in future studies. Measurement of signs and reporting of symptoms should be objectively recorded on admission by trained professionals as far as possible. Such an approach would enable a network meta-analysis to be undertaken with the aim of obtaining a parsimonious set of symptoms able to reliably diagnose EVD.

The current WHO case definition[7]for EVD does not include some of the symptoms found to be strongly predictive for EVD on this analysis, such as confusion, conjunctivitis, jaundice and (although not very prevalent in either group) cough. It also includes symptoms found here not to be predictive for EVD, including headache, joint pain and difficulty breathing, with the latter actually being more prevalent in the non-EVD group. The WHO case definition was reported in a recent meta-analysis (for all ages) to have a sensitivity of 81·5% (95% CI 74·1–87·2) and a low specificity of 35·7% (95% CI 28·5–43·6) [33]. Including a wider range of symptoms found to be predictive in this analysis, as well as omitting those both uncommon and not predictive, may help to improve the utility of such screening tools.

## Conclusions

The existing literature fails to provide a unified position on the symptoms most predictive of EVD. This analysis found that late presenting symptoms including confusion, conjunctivitis, dysphagia and jaundice, were highly predictive for EVD. In addition, early non-specific symptoms of diarrhoea, vomiting, fatigue, fever, muscle pain and cough, were also strongly predictive for EVD. Confirmation of these findings across datasets (or ideally an aggregation of them all) will aid effective future clinical assessment, risk stratification tools and emergency epidemic response planning.

## Supporting information

S1 Checklist PRISMA checklist. (DOC)

S1 Fig Estimated pooled odds ratio of symptoms in EVD compared to non-EVD. (PDF)

S1 Table Risk of bias criteria. (DOCX)

S1 Data Raw data file (included studies). (XLSX)
