# Supplementary figures and images for: Meta-analysis of predictive symptoms for Ebola virus disease
Source: PLoS Negl Trop Dis. 2020 Oct 23;14(10):e0008799. doi: 10.1371/journal.pntd.0008799 (PMC7641466; doi:10.1371/journal.pntd.0008799)

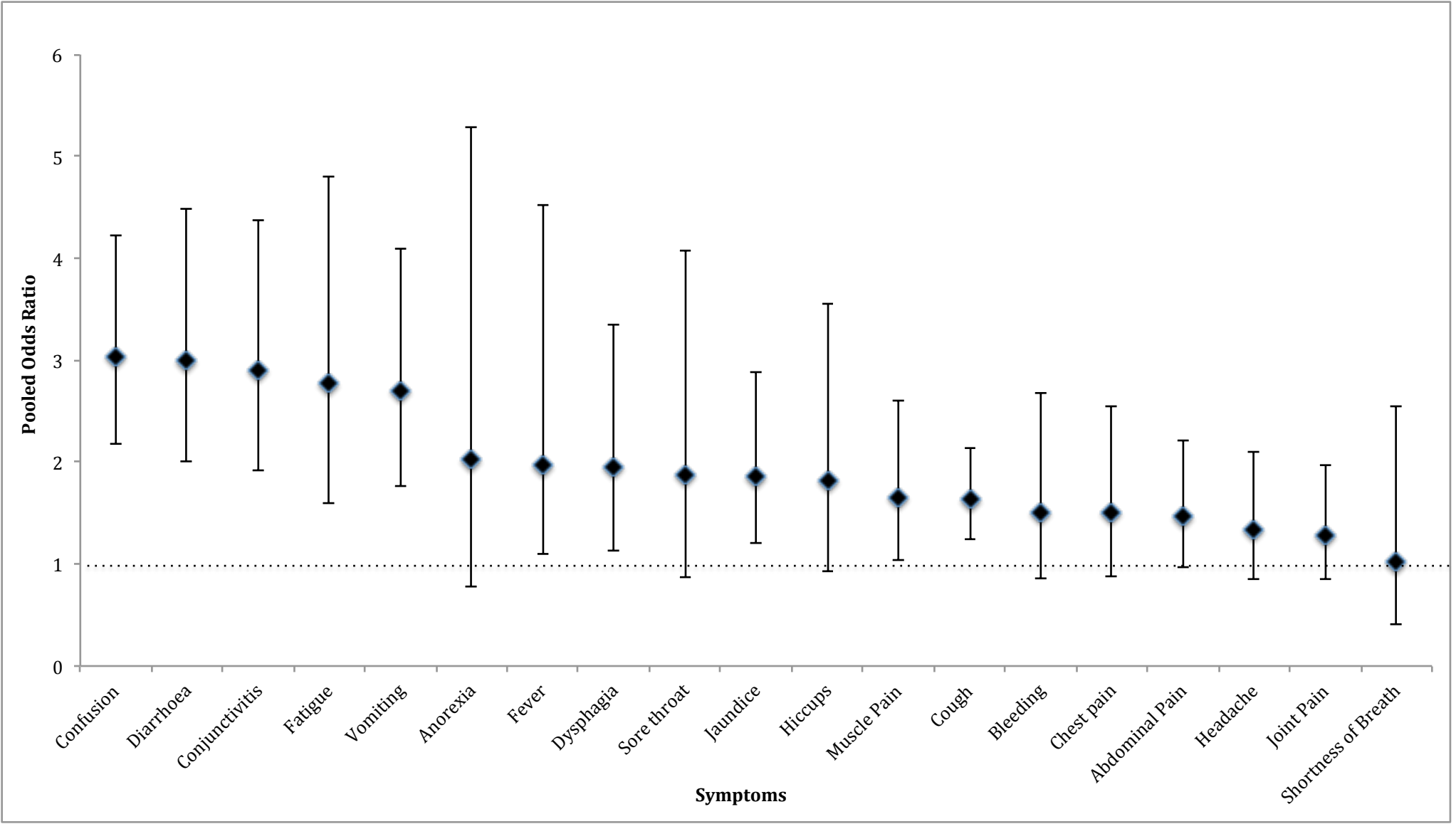

Supplement: S1 Fig — (PDF) [file pntd.0008799.s002.pdf]
