# Supplementary material for: Meta-analysis of predictive symptoms for Ebola virus disease
Source: PLoS Negl Trop Dis. 2020 Oct 23;14(10):e0008799. doi: 10.1371/journal.pntd.0008799 (PMC7641466; doi:10.1371/journal.pntd.0008799)
Supplement: S1 Table — (DOCX) [file pntd.0008799.s003.docx]

Risk of Bias Criteria

| Code | Bias | Description |
| --- | --- | --- |
| A | Selection | A clear definition of source population  Clear eligibility criteria for selection of subjects |
| B | Measurement | Symptoms recorded through a standardized, reliable and consistent method, on initial admission for all subjects  Data for all participants on medication/other medical conditions which could affect initial presentation  Objective assessments of symptoms made where possible |
| C | Detection | Outcomes clearly defined  The method of outcome assessment is valid and reliable |
| D | Reporting | Appropriate statistical analysis carried out and confidence intervals provided  Main potential confounders identified and taken into account |
| E | Other | Study addresses a clearly focussed question with a measurable outcome  A power calculation is reported  Data quality checks performed |
